# Inferential reasoning abilities in wild-caught bumblebees

**DOI:** 10.1098/rsbl.2023.0561

**Published:** 2024-06-12

**Authors:** Gema Martin-Ordas

**Affiliations:** ^1^ Division of Psychology, University of Stirling, Stirling, UK

**Keywords:** inferential reasoning, logical reasoning, invertebrates, bumblebees

## Abstract

The ability to make a decision by excluding alternatives (i.e. inferential reasoning) is a type of logical reasoning that allows organisms to solve problems with incomplete information. Several species of vertebrates have been shown to find hidden food using inferential reasoning abilities. Yet little is known about invertebrates’ logical reasoning capabilities. In three experiments, I examined wild-caught bumblebees’ abilities to locate a ‘rewarded’ stimulus using direct information or incomplete information—the latter requiring bees to use inferential reasoning. To do so, I adapted three paradigms previously used with primates—the two-cup, three-cup and double two-cup tasks. Bumblebees saw either two paper strips (experiment 1), three paper strips (experiment 2) or two pairs of paper strips (experiment 3) and experienced one of them being rewarded or unrewarded. At the test, they could choose between two (experiment 1), three (experiment 2) or four paper strips (experiment 3). Bumblebees succeeded in the three tasks and their performance was consistent with inferential reasoning. These findings highlight the importance of comparative studies with invertebrates to comprehensively track the evolution of reasoning abilities, in particular, and cognition, in general.

## 1. Introduction

Inferential reasoning is usually considered a uniquely human and language-dependent ability. However, when, for example, a reward is hidden in one of two containers (A or B)—without subjects knowing in which container the reward goes in, and one of them is shown to be empty (A)—2-year-old children [1,2] and different non-human animal (henceforth animals) species ([3] for a review) search for the reward in the other container (B)—the so-called two-cup paradigm [4]. Succeeding in this task is consistent with inferential reasoning by exclusion (i.e. ‘the reward is in A or B, not in A, therefore B’). However, alternative accounts suggest that successful performance could rely on non-deductive strategies. For example, when individuals see the container A being empty, they could avoid selecting A and, instead, select B only because it is the other possible container available (‘avoidance hypothesis’). That is, under this interpretation, individuals do not necessarily represent A and B as the locations where the reward could be hidden [2]. A second non-deductive strategy implies that individuals treat the containers as two possible locations where the reward can be hidden without representing the dependent relationship (‘or’) between them. Thus, when individuals see that A is empty, they select B based on their initial representation as potentially containing the reward (i.e. ‘maybe A and maybe B’ rather than ‘if A or B’) [2].

Two relatively recent studies control for these alternative explanations. In a *three-cup paradigm* with great apes [5], an experimenter hid a reward inside one of two containers that were placed behind a barrier. Therefore, this hiding event happened without subjects knowing in which of the two containers the reward had been placed. Importantly, there was also a third container that always remained visible to the subjects. When the barrier was lifted, subjects were shown one of the two containers previously placed behind the barrier (the experimenter always showed the empty one). The *avoidance hypothesis* would predict randomly choosing between the two containers whose content was not shown to the subjects. However, great apes consistently selected the second container that had been placed behind the barrier—which is congruent with an inferential reasoning strategy. To control for the second account, Mody & Carey [2] developed a *double two-cup paradigm*. Children experienced how two containers—in two sets of two (sets A and B)—were baited behind a barrier. This hiding event took place without subjects knowing where the reward was placed. Next, children saw that one of the containers in one of the sets (A) was empty and then had to choose one of the four containers. Only children older than 3 years of age inferred that selecting the second container in set A would warrant obtaining the reward—which is consistent with an inferential reasoning strategy.

Forms of logical reasoning have been demonstrated in invertebrates (e.g. [6–8]); however, it is still an open question whether inferential reasoning is present in animals other than vertebrates. To help close this gap, I tested wild-caught bumblebees in three inferential reasoning tasks previously used with primates: (i) two-strip paradigm (experiment 1), (ii) three-strip paradigm (experiment 2), and (iii) double two-strip paradigm (experiment 3). Since bumblebees have recently been shown to spontaneously reason by analogy [7], they are a good model to further investigate reasoning abilities. Investigating whether making inferences by excluding different alternatives extends to invertebrates is critical to understanding the structure of abstract reasoning. If a shared structure exists, it is expected that, like in vertebrates, bees are able of inferential reasoning.

## 2. Experiment 1: two-strip paradigm

I examined bees’ abilities to reason by exclusion to locate a ‘rewarded’^1^ strip based on the location of a non-rewarded strip [[4]]. Bees experienced either a rewarding (*direct cue*) or a non-rewarding paper strip (*exclusion cue*). Based on this information, the bees had to choose one of two strips when presented to them a second time. Since bumblebees can spontaneously reason about relations [7], they should also succeed in this task.

### (a) Methods

#### (i) Subjects

Thirty-three bees were captured in May 2023 (Stirlingshire, UK). Three bees completed less than six trials and were not included in the analyses. The final sample was 30 bees (table 1). Sex was visually identified (females = 30).

**Table 1.** Bee species and sample sizes included in each experiment. Note that different individuals were used for each experiment.

bee species	two-strip paradigm	three-strip paradigm	double two-strip paradigm
*Bombus pratorum*	*n* = 15	*n* = 9	*n* = 1
*Bombus pascuorum*	*n* = 8	*n* = 9	*n* = 12
*Bombus hypnorum*	*n* = 4	*n* = 4	—
*Bombus hortorum*	*n* = 3	—	*n* = 3
*Bombus terrestris complex*	—	*n* = 8	*n* = 14

#### (ii) Materials

A transparent plastic tube (14 × 3.5 cm) with two holes at one end (transparent end) through which the stimuli could be inserted was used. The distance between the holes was 1 cm. For experiments 1–3, yellow paper strips (3 × 0.2 cm) were used as stimuli, and they were introduced through the holes in the tube. The strips were fixed in playdoh to introduce them simultaneously in the tube.

#### (iii) Procedure

**Figure 1.**
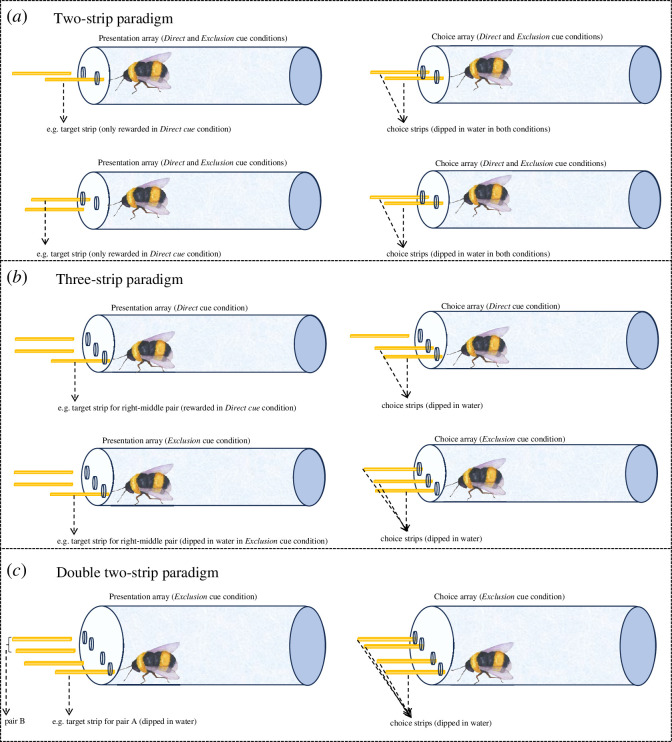
Representation of the experimental conditions (*Direct* and *Exclusion cues*) for the (*a*) *two-strip,* (*b*) *three-strip and* (*c*) *double two-strip paradigms*; experiments 1, 2 and 3, respectively. Bees faced two (experiment 1), three (experiment 2) or four objects (experiment 3) and experienced one of them dipped in sucrose (*Direct cue* condition) or in water (*Exclusion cue* condition). The bees’ task was to search among the objects when presented a second time (Choice array). For each experiment, the position of the target strip is counterbalanced across trials—as represented in (*a*). In experiment 2 (*b*), the pair of strips that contained the target strip was counterbalanced across subjects. In experiment 3 (*c*), for each subject, the target strip could be in pair A or B, and this was counterbalanced across trials. See electronic supplementary material, figure S1 for a representation of the *Familiarization* and *Control* conditions of experiments 2 and 3.

The electronic supplementary material provides general information on how bees were captured as well as on methods for experiments 1–3. The experimental procedure for experiment 1 consisted of two steps (figure 1*a*): (i) presentation. First, bees were presented with two strips but only one of them—target strip—was introduced through one of the tube holes (e.g. left). The other strip was left outside the tube—far enough (e.g. 1 cm from right hole) so bees could see it but could not touch it; and (ii) choice. Once bees touched the target strip (bees were given, on average, 4–5 s to drink the reward and this also applied to experiments 2 and 3), experimenter (E) removed it and introduced two strips dipped in water when the bees were 1.5–2 cm away from the stimuli. A choice was considered when bees touched the strip with the antennae or proboscis.

There were two conditions always presented in the following order: (i) *Direct cue* (six trials): E presented bees first with the strip dipped in 50% (w/w) sucrose and (ii) *Exclusion cue* (two trials): E presented bees first with the strip that was not baited. In both conditions, after the presentation of the target strip, bees were presented with two unrewarded strips for them to make a choice. Since both steps of the *Exclusion cue* trials were unrewarded, bees were only presented with the minimum number of trials to warrant that the target strip was presented in the two locations and to ensure that lack of motivation did not affect bees’ performances. This logic was also followed for the *Exclusion cue* trials in experiments 2 and 3. Which strip—left or right—was used as the target was counterbalanced across trials. For experiments 1–3, the inter-trial intervals were approximately 2 min and during this time, subjects were allowed to freely move in the tube. New materials were used for both steps within a trial and for each trial. Bees did not receive any training prior to these trials.

#### (iv) Analyses

Data were analysed using R version 2023.12.1+402 using a binomial general linear mixed model (GLMM) [9]. The dependent variable was whether bees’ choice in the *Direct cue* and *Exclusion cue* trials was correct (coded 1) or incorrect (coded 0), the independent variable was conditioned as a categorical variable and a random factor was the individual bees. A second model was run including bee’s choice as a dependent variable, condition as an independent variable and individual bees, trial number and species as random factors (see the electronic supplementary material). This model was also run for experiment 2. In both cases, this analysis was done to assess whether trial number and bee species influenced subjects’ performances. In the *Direct cue* trials, selecting the strip in the location where the previously rewarded strip had been found was correct. In the *Exclusion cue* trials, selecting the strip in the alternative location to where the previously non-rewarded strip had been found was correct. Wilcoxon tests and binomial tests were used to analyse if performance was significantly above chance. Values of *p* below 0.050 were considered to provide significant differences.

### (b) Results and discussion

**Figure 2.**
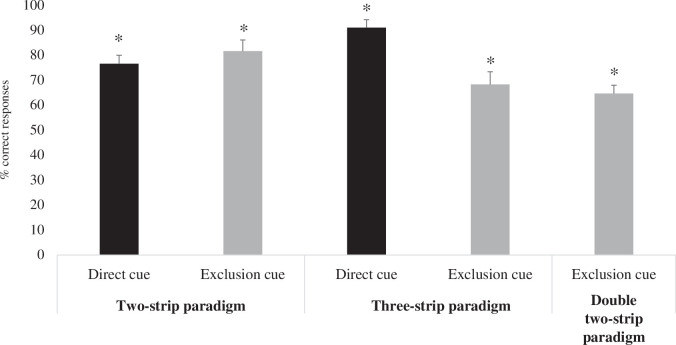
The percentage of correct responses in the *Direct cue* and *Exclusion cue* conditions of experiment 1 (two-strip paradigm) and experiment 2 (three-strip paradigm), respectively. For experiment 3 (double two-strip paradigm), only the percentage of correct responses in the *Exclusion cue* condition is represented. The percentage of correct responses was calculated out of the trials performed for each bee. The individual percentages were then used to calculate the group mean. The asterisk indicates the conditions in which bees performed significantly above chance. The bars represent the s.e.m.

Condition did not affect bees’ performance (estimate s.d. = −0.200, *z* = −0.539, *p* = 0.59, 95% CI = −0.963 to 0.505; figure 2). Bees chose the correct strip significantly above chance in the first trial of the *Direct cue* (*p *< 0.001) and *Exclusion cue* conditions (*p* = 0.005). Overall, subjects also performed significantly above chance in the *Direct cue* (*W* = 276, *p* < 0.001) and *Exclusion cue* conditions (*W* = 190, *p* < 0.001).

Bees selected the correct strip in both conditions, and they did so from trial 1. These results suggest that bees can infer the location of the ‘rewarded’ strip when only partial information is provided. However, performance in this task is also consistent with the ‘avoid the non-rewarded strip’ strategy [10]. Thus, it is plausible that bees might have simply selected the alternative strip to the non-rewarded one without expecting the other strip to be rewarded. To test this possibility, I conducted the next experiment in which avoiding the non-rewarded strip would lead to random decisions between two alternative strips.

## 3. Experiment 2: three-strip paradigm

Bees faced three strips that were always visible to them and experienced that only one of them (e.g. left) within a particular pair (e.g. left-middle strips) was rewarded. In the experimental trials (i.e. *Exclusion cue*), however, bees were always presented with the non-rewarded strip within the pair and, next, had to choose between three strips. This experiment offers two alternative strips to the non-rewarded strip—allowing examination of whether bees prefer the strip that might contain the reward (the alternative strip in the pair) or the strip where the reward is unknown. If bees are following the heuristic ‘avoid the non-rewarded strip’, they would choose randomly between the two alternative strips. However, if logical reasoning underlies their performance, bees will show a preference for the alternative strip in the pair.

### (a) Methods

#### (i) Subjects

Thirty-four bees were captured in June 2023 (Stirlingshire, UK). Four bees completed less than six trials and were not included in the analyses. The final sample was 30 bees (table 1): 29 females and one could not be clearly identified.

#### (ii) Materials

A transparent plastic tube (14 × 3.5 cm) with three holes (0.5 cm between holes) at one end (transparent end) through which the stimuli could be inserted was used.

#### (iii) Procedure

There were four different conditions (*n* = 10 trials). For the *Direct cue* and *Exclusion cue* trials, the experimental procedure consisted of the same two steps as in experiment 1. For the *familiarization* and *control* trials, there was only one step in which bees were presented with the stimuli once (figure 1*b* and electronic supplementary material, figure S1*a*). These conditions were always presented in the following order: (i) *familiarization* (three trials). Bees were presented with three strips—with only one of them dipped in sucrose—and were allowed to explore the three strips. This was done to let the bees experience that only one of the strips was rewarded. For 47% of the bees, the rewarded pair was middle-left strips, and for the other 53%, the middle-right strips. Each trial ended when bees explored the three strips. This also applies to experiment 3. (ii) *Direct cue* (three trials). Bees were presented with three strips and only the rewarded target strip was introduced through one of the tube holes (e.g. left). Once the bees experienced the target strip, E removed it and introduced two strips dipped in water (e.g. left and middle)—the third one (e.g. right) was left outside the tube (e.g. 1 cm from right hole) so bees could see it but not touch it. This was done to further familiarize bees with the location of the reward (e.g. left or middle strip) within a pair (e.g. left-middle). (iii) *Exclusion cue* (two trials). Bees first experienced an unrewarded target strip (e.g. left). Once the bees experienced the target strip, E removed it and presented the bees with three strips dipped in water for them to make a choice. (iv) *Control* (two trials). Bees were presented with three strips dipped in water to examine whether bees preferred any of the locations.

#### (iv) Analyses

The same analyses as in experiment 1 were conducted. A binomial GLMM for the *Control* phase was also conducted. The dependent variable was bees’ strip choice (coded 1 or 0), the independent variable was the location of the strip (left, middle and right) and a random factor was the individual bees. See electronic supplementary material for the coding of bees’ responses.

### (b) Results and discussion

Condition had an effect on bees’ performance (estimate s.d. = 1.558, *z* = 3.36, *p* < 0.001, 95% CI = 0.681 to 2.515; figure 2)—bees performed better in the *Direct cue* condition than in the *Exclusion cue* condition. Bees chose the correct strip significantly above chance in the first trial of the *Exclusion cue* (*p* = 0.006) and *Direct cue* conditions (*p* < 0.001). Bees also performed significantly above chance in both conditions (*Exclusion cue: W* = 447, *p* < 0.001; *Direct cue: W* = 458, *p* < 0.001). When making mistakes (*n* = 19) in the *Exclusion cue* condition, bees incorrectly chose the third strip in only 15% of the trials.

In the *Control* condition, bees selected the left strip (40% of the trials) more often than the middle (22%) one (estimate s.d. = −0.870, *z* = −2.03, *p* = 0.041, 95% CI = −1.73 to −0.04) and no differences between right (38%) and middle strips were found (estimate s.d. = −0.076, *z* = −0.195, *p* = 0.845, 95% CI = −0.846 to 0.691). Although, subjects seemed to show a preference for the outside strips.

After experiencing the non-rewarded strip, bees selected the alternative strip of the pair. Bees also selected the ‘rewarded’ strip in the *Direct cue* condition and they did so more often than in the *Exclusion cue* condition. This could be because in the *Direct cue* condition bees only had to choose between two strips. Importantly, bees’ preference for the correct strip was already apparent in the first trial of both conditions. An alternative explanation is that subjects reasoned that the reward may be, for example, on the left strip without necessarily connecting the different alternative locations [2]. To test this, I next presented bees with the *double two-strip* paradigm, in which understanding the link between two sets of strips is required to succeed.

## 4. Experiment 3: double two-strip paradigm

Bees faced two pairs of strips (e.g. A and B) and experienced that only one strip within each pair was rewarded (e.g. A: left; B: right). At test, bees were always presented with only one unrewarded strip (e.g. left) of one pair (e.g. A). If bees reason by exclusion, they should combine the information about the unrewarded strip with the representation of the potential location of the ‘rewarded’ strip (pair A: left or right) to infer the correct strip (pair A: right). However, if bees consider the strips independently from each other, experiencing the unrewarded strip would eliminate that strip as a potential choice, but the remaining three could be equally good choices [5].

### (a) Methods

#### (i) Subjects

Thirty-one bees were captured in June 2023 (Stirlingshire, UK). One bee completed less than six trials and was not included in the analyses. The final sample was 30 bees (table 1): 29 females and one male.

#### (ii) Materials

A transparent plastic tube (11 × 4.5 cm) with two sets of two holes was used: two of the holes (4 mm between them) were drilled closer to the left side of the tube and the other two (4 mm between them) closer to right side. The distance between the sets of holes was 1.5 cm.

#### (iii) Procedure

There were two different conditions (*n* = 8 trials). As in experiment 2, for the *Exclusion cue* trials, there were two steps (i.e. presentation of target strip followed by the presentation of two pairs of two strips) and for the *Familiarization* trials only one (i.e. presentation of two pairs of two strips; see figure 1*c* and electronic supplementary material, figure S1*b*). The two conditions were always presented in the following order: (i) *Familiarization* (four trials). Bees were presented with two pairs of two strips (i.e. pair A: left, right; pair B: left, right). Bees could explore the four strips to experience that only one strip in each pair had sucrose; (ii) *Exclusion cue* (four trials). Only one of the four strips—always an unrewarded strip—was introduced through the tube (e.g. left from pair A). Once bees touched the strip, E removed it and introduced four unrewarded strips for them to make a choice.

#### (iv) Analyses

Binomial and Wilcoxon tests were conducted, and chance levels were set at 50% [11]. The electronic supplementary material includes a Kruskal–Wallis test to examine species differences. See also the electronic supplementary material for the coding of bees’ responses.

### (b) Results and discussion

Bees chose the correct strip significantly above chance in the first trial of the *Exclusion cue* condition (*p* = 0.005). Subjects also performed significantly above chance in this condition (*W* = 190, *p* < 0.001; average performance 64.7%; figure 2). These results suggest that bees inferred that the alternative strip within the pair to the target one must contain the reward and chose this option.

## 5. General discussion

Bees’ performance is consistent with inferential reasoning. Experiments 2 and 3 suggest that neither the ‘avoid the non-rewarded strip’ nor the ‘maybe A, maybe B’ accounts can explain the present results.

First trial success precludes learning as a mechanism driving bees’ choices [12]. Performance in experiment 3 also indicates that bees could have understood that the probability of finding the ‘rewarded’ strip was related to a particular pair of strips and not to the remaining strips. It is possible, then, that bees represented a disjunctive relation (‘or’) between the strips within a pair. An alternative explanation is local enhancement [13]; that is, by experiencing that one of the strips within the pair is non-rewarded, bees might have focused their attention on that particular pair, which might have facilitated bees selecting the alternative strip within that pair. Note that the strips were introduced when the bees were not close to the strips to avoid potential biases. Moreover, if local enhancement accounts for bee performance, in experiment 2, bees would have randomly selected between the outside strips when the middle strip was unrewarded. However, this is not what they did (i.e. correct outside strip: 64%; incorrect outside strip: 23%; middle strip: 13%).

Studies have demonstrated that bees possess logical reasoning abilities (e.g. [6,7]). The current results extend previous findings by showing that bees are able of inferential reasoning and might be forming an expectation about the location of the ‘rewarded’ strip once they experience an unrewarded one. When navigating their physical world, bees face situations where the information necessary for making a decision could be incomplete or lacking. For example, when deciding whether to visit a flower, bees might infer that if no scent marks have been deposited in the flower, then the flower might well contain nectar. Using the available information to infer information that is missing can be beneficial in these situations. Importantly, the results presented here are along the lines of previously reported findings in non-human primates (e.g. [5,14], a parrot [15] and young children [2]).

To conclude, bumblebees select a correct option by excluding alternative ones—adding to previous evidence showing bee complex cognitive abilities. Studies like the ones presented here indicate that small-brained social insects are useful models for understanding the evolution of cognition.

## Data Availability

Data and R code are available from the Dryad Digital Repository [16]. Electronic supplementary material is available online at [17].

## Footnotes

^1^
Rewarded written in quotation marks (i.e. ‘rewarded’) refers to the strip that should contain sucrose but that it does not. The strip was dipped in water in order to avoid external cues to facilitate bees’ choices. See §2.
